# Joint Goals in Older Couples: Associations With Goal Progress, Allostatic Load, and Relationship Satisfaction

**DOI:** 10.3389/fpsyg.2021.623037

**Published:** 2021-04-20

**Authors:** Nadine Ungar, Victoria I. Michalowski, Stella Baehring, Theresa Pauly, Denis Gerstorf, Maureen C. Ashe, Kenneth M. Madden, Christiane A. Hoppmann

**Affiliations:** ^1^Department of Psychology, University of British Columbia, Vancouver, BC, Canada; ^2^Department of Psychology, Humboldt University Berlin, Berlin, Germany; ^3^Center for Hip Health and Mobility, Vancouver, BC, Canada; ^4^Department of Family Practice, University of British Columbia, Vancouver, BC, Canada; ^5^Department of Medicine, University of British Columbia, Vancouver, BC, Canada

**Keywords:** close relationships, joint goals, older adults, relationship satisfaction, allostatic load, goal progress, couple, health

## Abstract

Older adults often have long-term relationships, and many of their goals are intertwined with their respective partners. Joint goals can help or hinder goal progress. Little is known about how accurately older adults assess if a goal is joint, the role of over-reporting in these perceptions, and how joint goals and over-reporting may relate to older partners' relationship satisfaction and physical health (operationally defined as allostatic load). Two-hundred-thirty-six older adults from 118 couples (50% female; *M*_age_ = 71 years) listed their three most important goals and whether they thought of them as goals they had in common with and wanted to achieve together with their partner (self-reported joint goals). Two independent raters classified goals as “joint” if both partners independently listed open-ended goals of the same content. Goal progress and relationship satisfaction were assessed 1 week later. Allostatic load was calculated using nine different biomarkers. Results show that 85% self-reported at least one goal as joint. Over-reporting– the perception that a goal was joint when in fact it was not mentioned among the three most salient goals of the spouse – occurred in one-third of all goals. Multilevel models indicate that the number of externally-rated joint goals was related to greater goal progress and lower allostatic load, but only for adults with little over-reporting. More joint goals and higher over-reporting were each linked with more relationship satisfaction. In conclusion, joint goals are associated with goal progress, relationship satisfaction, and health, but the association is dependent on the domain of functioning.

## Introduction

Many marriages in old age are long-term relationships (Meegan and Berg, 2002). Older spouses tend to become more central to each other due to an increased focus on close, emotionally meaningful relationships (Baltes and Carstensen, 1999). Partners shape each other's behavior, physiology, and health (e.g., review by Kiecolt-Glaser and Wilson, 2017). One underlying mechanism may be shared goals (Lauer et al., 1990; Berg and Upchurch, 2007). Goals serve as a personal compass into old age (Hooker, 2002). Goals are mostly examined in samples of unrelated individuals, and yet, they often need to be coordinated with close others such as spouses (Baltes and Carstensen, 1999; Mann et al., 2013; Fitzsimons and Finkel, 2018). Little is known about the proportion and type of goals that older spouses have in common and the correlates for everyone involved. Using a sample of 118 older couples, this brief report seeks to fill this gap by investigating the joint nature of everyday goals, taking into account the perspective of both partners as well as potential implications for goal progress, health, and relationship satisfaction.

### Joint Goals and Goal Progress

We operationally define goals as joint when spouses report goals they have in common and want to achieve together with their partner. Extending research with younger couples and a focus on relationship goals (Avivi et al., 2009) we assume that a higher number of joint goals allows older spouses to pool their resources and make better goal progress.

Importantly, partner ratings of a goal as joint might not always be accurate; instead, they may be positively biased (Martz et al., 1998; Gagné and Lydon, 2004). Known as the “positivity effect,” older adults prioritize positive over negative information (Baltes and Carstensen, 1999; Carstensen et al., 2003). Older adults also view their spouse's behavior as more positive during conflict than middle-aged couples in the eye of independent observers (Story et al., 2007). We, therefore, assume that perceptions of goals as joint may not always be accurate but positively biased in the present sample. In other words, older spouses may over-report joint goals relative to external raters. Over-reporting occurs if older adults—thinking of joint goals as joint when in fact it is no mentioned among the three most salient goals of the spouse. In line with the Transactive-Goal-Dynamics Theory (Fitzsimons and Finkel, 2018) and previous research indicating that older couples with more joint goals use more collaborative problem-solving (Hoppmann and Gerstorf, 2013) and engage in more spousal goal involvement (Meegan and Goedereis, 2006), we argue that to translate joint goals into action, goal coordination between partners is key. To collaboratively engage in goal coordination, the Transactive-Goal-Dynamics Theory states that it is necessary that partners adjust their behavior to each other's goal-relevant states (e.g., expectations). A discrepancy between assumed and actual joint goals makes that difficult and may thus hinder goal coordination and ultimately goal progress. We thus assume that joint goals can be better pursued if both partners have correct insights into each other's salient goals, which facilitates goal coordination. Therefore, we expect that more joint goals are associated with greater goal progress, particularly when older adults accurately perceive their salient goals as jointly held, i.e., they engage in little over-reporting.

### Joint Goals and Allostatic Load

Joint goals may not only impact everyday behaviors but also shape health outcomes, possibly through stress-related pathways and lifestyle factors (Hoppmann and Klumb, 2006; Hoppmann and Gerstorf, 2014; Feeney and Collins, 2015). This may be particularly true among older adults due to age-related wear and tear (Seeman and Gruenewald, 2006). A well-established index of stress-related wear-and-tear is allostatic load (Seeman et al., 2004). Allostatic load taps into different biological systems, including neuroendocrine and cardiovascular risk markers (Seeman and Gruenewald, 2006). Previous research indicates that social factors such as positive and close relationships, spousal presence and social support are linked to reduced allostatic load (Seeman et al., 2002; Juster et al., 2010; Brooks et al., 2014; Priest et al., 2015). In contrast, higher spouse and family negativity are related to higher allostatic load (Brooks et al., 2014). We expected that a high number of joint goals would be associated with low allostatic load, possibly because older spouses with many joint goals are better able to coordinate complex health goals, engage in dyadic planning, and accordingly have healthier lifestyles and experience less stress (Keller et al., 2017; Wiley et al., 2017; Berli et al., 2018; Fitzsimons and Finkel, 2018). Thus, we expect that when older adults' perceptions of joint goals converge with what independent raters are able to detect, they can better coordinate goal-directed activity. Therefore, parallel to our hypothesis regarding goal progress, we expect the association between joint goals and low allostatic load to be more pronounced if older adults accurately perceive their goals as jointly held, i.e., they engage in little over-reporting.

### Joint Goals and Relationship Satisfaction

With increasing age and a limited future time perspective, there is a shift in goals toward emotionally meaningful social relationships (English and Carstensen, 2014). Therefore, we aimed to investigate - as a third relevant correlate - how joint goals might be related to relationship satisfaction. The Eudaimonic Theory of Marital Quality proposes that shared goals are central ingredients of marital satisfaction (Fowers and Owenz, 2010). Shared goals address inherent needs for security and belonging and foster dyadic processes such as the inclusion of the partner in the self, couple identity, and commitment. We assume that rosy-colored views of goals as shared with a partner would be positively associated with relationship satisfaction and that this association would not be tempered by positively biased over-reporting of joint goals as relationship satisfaction may be based on subjective perceptions and is less behavioral than the other two indicators. Accordingly, we expected that more joint goals would be associated with higher relationship satisfaction.

### The Current Study

Incorporating the perspective of both spouses, this brief report elucidates how joint goals are linked with goal progress, health, and relationship satisfaction taking into account meaningful differences between subjective and external ratings of joint goals. Specifically, we hypothesized that more joint goals would be related to greater goal progress (hypothesis 1, Hp1), lower allostatic load (Hp2), and higher relationship satisfaction (Hp3). In line with motivational theories, we assumed that goal coordination is necessary to translate joint goals into goal progress and allostatic load. Thus, we expected the associations between joint goals and goal progress and allostatic load to be stronger if older adults accurately perceived their salient goals as jointly held (moderation effect).

## Methods

### Participants

Participants were 118 community-dwelling couples (*N* = 236 individuals). From the original 258 participants who entered the study, nine couples dropped out after the baseline session and two further couples had to be excluded due to missing values on the main outcome variables. The sample included ethnically diverse heterosexual couples aged 60–87 years (*M* = 71.01*, SD* = 5.97) as described in Table 1. 82.4% of couples were married and 7.6% lived in a domestic partnership; relationship duration was 41.01 years on average (*SD* = 13.30).

**Table 1:** Descriptive statistics and correlations of sample characteristics (*N* = 236) and study variables.

	**Variables**	**Women**		**Men**		**Correlations**						
		***M***	***(SD)***	***M***	***(SD)***	**1**	**2**	**3**	**4**	**5**	**6**	**7**
1	Age	69.77	5.44^d^	72.26	6.22^d^	0.66***	−1.70	−0.01	0.05	−0.01	0.10	0.02
2	Self-rated health	3.27	0.96	3.25	0.95	−1.60	0.39^***^	0.06	−0.12	0.41^***^	−0.05	0.03
3	Goal progress^a^	3.15	0.87	3.06	0.99	−0.60	0.15	**0.07**	0.17	−0.10	0.05	0.05
4	Allostatic load^b^	1.00	0.80	1.15	0.93	0.04	−0.10	−0.12	**0.09**	−0.08	−1.50	−1.40
5	Relationship satisfaction^c^	4.06	0.80^d^	4.29	0.57^d^	−0.18	0.40^***^	0.07	−0.03	0.48^**^	0.10	0.03
6	Number of joint goals (self-report)	1.58	1.09^d^	1.97	0.98^d^	−0.05	0.01	0.22^*^	0.07	0.40^***^	0.30^***^	0.58^***^
7	Over-reporting of joint goals	0.88	0.88^d^	1.19	0.95^d^	−0.11	0.20	0.06	−0.01	0.25^**^	0.60^***^	0.31^***^
		***N***	***%***	***N***	***%***							
Ethnicity												
	Caucasian/White	71	60.2	70	59.8							
	Asian	39	33.1	42	35.9							
	Aboriginal	2	1.7	0	0							
	Hispanic	1	0.8	1	0.9							
	Other	5	4.2	4	3.4							
English language^e^		69	58.5	69	58.5							
University education or equivalent		82	70.1	79	66.9							
Retired		106	90.6	100	85.5							

a range from 1 (none) to 5 (a lot)

b range from 0 (very low risk for chronic disease) to 4 (very high risk for chronic diseases)

c *range from 1 (very low relationship satisfaction) to 5 (very high relationship satisfaction)*;

d *mean differences between men and women are significant*;

e *language of study participation (English vs. Mandarin); correlations of women are presented above the main diagonal, correlations of men are presented below the main diagonal, and correlations between men and women are displayed in bold in the main diagonal*.

* *p < 0.05*.

** *p < 0.01*,

*** *p < 0.001*.

### Procedure

The study was part of a larger project on spousal health dynamics (described in Supplementary Material 1 and Pauly et al., 2019). Couples were recruited in the greater Vancouver area using various strategies (e.g., media, community organizations). Informed consent was obtained (University of British Columbia ethics board), and each partner received $100 compensation. The study consisted of a baseline-session, a 1-week time-sampling phase, and an exit-session 1 week later.

### Measures

#### Personal Goals and Self-Reported Joint Goals

Participants reported three open-ended, particularly salient goals (A,B,C) whose realization was highly important to them within the upcoming week (based on Hoppmann and Klumb, 2006; see Supplementary Material 2). Afterward, participants self-rated their goals along 12 domains (e.g., “work,” “family,” multiple answers possible). For each goal, participants were asked, “Is this a goal that you and your partner have in common and want to achieve together?.” This measure was used to calculate the self-reported number of joint goals (range 0–3) with a mean of *M* = 1.77, *SD* = 1.05.

#### Externally-Rated Joint Goals

Participants' three salient goals were also rated by two independent raters (NU and SB). They classified the goals as joint (=both partners mentioning the same goal) or individual (=goal was only mentioned by one partner) using a prior developed coding scheme (see Supplementary Material 3). For example, a goal was rated as joint if both partners mentioned the same activity, the same place, or the same third person. A goal was additionally rated as joint if one goal represented a subcategory of the other (e.g., “cleaning the house” and “cleaning the kitchen”). It was rated as individual if goals involved distinct activities (e.g., “swimming” vs. “tennis”).

Agreement between the independent raters was high (645 of 708 goals; 91.10%). In case of disagreement, a consensus was achieved during a discussion. Interrater-reliability was substantial (Cohen's Kappa = 0.794). The calculation of Cohen's Kappa is conservative in our case because it does not account for the order of potential goal combinations.

#### Over-Reporting of Joint Goals

To calculate over-reporting, externally-rated joint goals were compared to self-reported joint goals. If participants reported that they wanted to achieve a goal together with their partner, but the partner did not mention this goal, it was classified as “over-reported.” All other combinations counted as “not over-reported”^1^. For each participant, over-reporting was added up across all three goals. Thus, “over-reporting” ranged from 0 to 3 with a mean of *M* = 1.04, *SD* = 0.92.

#### Goal Progress Questionnaire

At the exit session, participants rated their goal progress since the baseline session, i.e., over the last week. For each goal separately, participants rated (1) their goal progress and (2) the extent to which they had reached that goal (1 = *none* to 5 = *a lot; M* = 3.11, *SD* = 0.93).

#### Allostatic Load

Allostatic Load was calculated as a sum score taking four different physiological systems into account (Seeman and Gruenewald, 2006; Chen et al., 2012): cardiovascular functioning (systolic and diastolic blood pressure), inflammation (C-reactive protein), lipid and general metabolic activity (body mass index, waist and hip circumference, lipid profile, HbA1C), and hypothalamic pituitary adrenal (HPA) axis activity^2^ (cortisol, calculated as area under the curve, Pruessner et al., 2003). An individual received a “1” per indicator if their value fell into the highest-risk quartile within the present sample (in the case of multiple indicators per system, the mean was used). Scores ranged from 0 to 1 (0 = no biomarker within the system in risk quartile; 1 = all biomarkers within the system within risk quartile). The allostatic load index was computed as the sum of the four systems with possible scores ranging from 0 to 4 (*M* = 1.07, *SD* = 0.87).

#### Relationship Satisfaction

Relationship satisfaction was assessed by the relationship assessment scale (Hendrick, 1988; Hendrick et al., 1998). Participants rated seven items (e.g., “How well does your partner meet your needs?”) from not at all (1) to very much (5); Cronbach's α = 0.89 and *M* = 4.17, *SD* = 0.70).

### Statistical Analyses

Hierarchical linear 2-level random intercept models for the outcomes (1) goal progress, (2) relationship satisfaction, and (3) allostatic load were conducted using the R package lme4 (Bates et al., 2014). Predictor variables were (1) the number of joint goals (externally-rated) at the couple-level, (2) over-reporting at the individual-level, and (3) the interaction between the two. The interaction was decomposed by calculating simple slopes (Preacher et al., 2006). Gender, age, language of study participation (English vs. Mandarin), and self-rated health (“poor = 1” to “excellent = 5”) served as control variables. All continuous variables were grand-mean centered and *R*^2^ is reported (see Supplementary Material 1 for analytic details).

## Results

### Descriptive Statistics for Joint Goals

External ratings identified one-third of goals (30.9%) as joint, with 65.3% of participants having at least one goal in common with the partner. Self-reports identified two-thirds of goals as joint (60.1%), twice as many as the external ratings suggest. Most participants (85.2%) self-reported at least one joint goal. Almost one-third (31.4%) reported wanting to achieve all three goals together with their partner. The difference between self-reports and external ratings was positively biased, and common: 65.6% of participants over-reported at least one joint goal. Men were older, more satisfied with their relationship, and reported more joint goals than women. No gender differences were found for goal progress and allostatic load (see Table 1).

Most goals were in the health domain (54.4%), followed by social (53.2%), work and finances (42.0%), and leisure (39.8%). Health-related goals had the highest proportion of joint goals (39.5% based on external ratings), whereas work and finance goals had the lowest proportion of joint goals (22.7%; see Table 2).

**Table 2:** Domains of goals and the proportion of joint goals.

**Domain**	**% of goals falling into this domain**	**Example goals**	**% of joint goals**		***X*^**2**^**	***p***
			**Within this domain**	**Outsidethis domain**^**a**^		
Health	54.4	“do a full health check”, “walk at least 15 minutes a day”	39.5	20.7	28.915	<0.001
Social	53.2	“invite family and friends for dinner”	31.8	29.9	0.300	n.s.
Work and finances	42.0	“paperwork for taxes”	22.7	38.8	15.772	<0.001
Leisure	39.8	“taking photos”	31.8	30.4	0.162	n.s.
Home management	25.3	“clean out closet and pictures”	30.3	31.1	0.038	n.s.
Other	40.8		27.3	33.3	2.247	n.s.

*N = 708 goals. Domains are based on participant's self-reports, and multiple answers were possible (i.e., one goal can belong to multiple domains). Percent joint is based on external rating. Domains were summarized as follows: “health”: health, physical activity; “social”: partnership, family, friends; “work and finances”: work and productive activities, finances; “other”: cognition and memory, volunteer, other; n.s., non significant*.

a *All goals, which do NOT belong to this domain*.

Self-reported and externally-rated joint goals were positively correlated (*r* = 0.29, *p* < 0.001). The number of joint goals (self-reported and externally-rated) was not significantly correlated with goal progress or allostatic load. Relationship satisfaction was positively associated with self-reported joint goals (*r* = 0.24, *p* < 0.001) and over-reporting (*r* = 0.15, *p* = 0.02). Intraclass correlations (ICC) ranged from 0.071 (goal progress) over 0.087 for allostatic load to 0.480 (relationship satisfaction), indicating that most of the variance was at the individual level.

### Goal Progress (Hp1)

**Figure 1:**
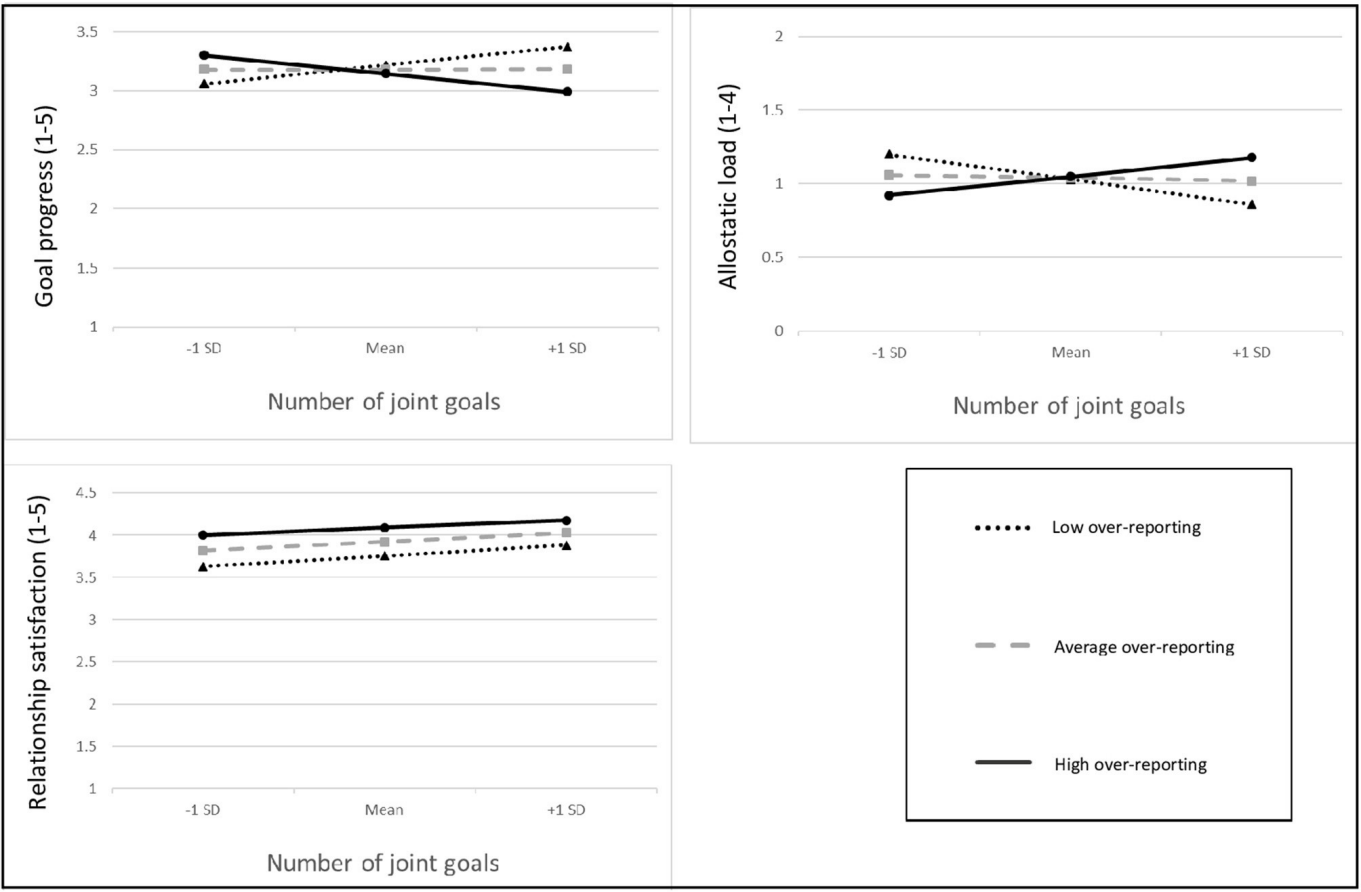
Interactions between the number of joint goals and over-reporting in explaining goal progress (upper left-hand panel), allostatic load (upper right-hand panel), and relationship satisfaction (lower left-hand panel). A larger number of joint goals is related to increased goal progress and lower allostatic load, but only for participants with few over-reporting. In contrast, a higher number of joint goals and more over-reporting are generally linked to higher relationship satisfaction.

Results regarding Hp1 concerned a moderation of over-reporting and joint goals on goal progress (Table 3). Control variables showed significant main effects for self-rated health (*b* = 0.16, *SE* = 0.07, *p* = 0.020) and English as language of study participation (*b* = −0.33, *SE* = 0.14, *p* = 0.019). No main effects emerged for over-reporting or number of joint goals. As hypothesized, the interaction between over-reporting and the number of externally-rated joint goals was significant (*b* = −0.20, *SE* = 0.09, *p* = 0.035; see Figure 1). More joint goals were related to more goal progress, but only when over-reporting was low; the simple slope for low over-reporting (1 *SD* below the mean) was 0.19 (0.08), *t* = 2.21, *p* = 0.028; the simple slope for average over-reporting (mean) was 0.001 (0.07), *t* = 0.01, *p* = 0.993 and the simple slope for high over-reporting (1 *SD* above the mean) was −0.19 (0.12), *t* = 1.49, *p* = 0.138. The model explained 13.99% of the variance.

**Table 3:** Multilevel analysis with goal progress, relationship-satisfaction, and allostatic load as outcome variables (*N* = 118 couples).

	**Goal progress**			**Allostatic load**			**Relationship satisfaction**		
	**Coefficient *(b*)**	***SE***	***p-*value**	**Coefficient *(b*)**	***SE***	***p-*value**	**Coefficient *(b*)**	***SE***	***p-*value**
**Intercept**	3.185^***^	0.215	<0.001	1.056^***^	0.161	<0.001	4.03^***^	0.085	<0.001
**Level 1 (person)**									
Over-reporting of joint goals	0.016	0.080	0.841	−0.042	0.074	0.568	0.132^*^	0.050	0.009
Age (in years)	−0.011	0.011	0.328	0.011	0.010	0.259	0.002	0.008	0.200
Gender	0.097	0.118	0.414	−0.156	0.109	0.154	−0.181^**^	0.065	0.007
Self-rated health	0.160^*^	0.068	0.020	−0.131^*^	0.063	0.040	0.168^***^	0.044	<0.001
English language^a^	−0.327^*^	0.139	0.019	0.271^*^	0.129	0.040	0.370^***^	0.960	<0.001
**Level 2 (couple)**									
Number of joint goals^b^	−0.049	0.093	0.595	0.000	0.021	0.999	0.160^*^	0.085	0.016
**Interaction**									
Over-reporting x number of joint goals	−0.196^*^	0.092	0.035	0.184^*^	0.086	0.033	−0.048	0.061	0.431
**Additional information**									
ICC	0.071			0.087			0.480		

*SE, standard error, goal progress ranged from 1 (none) to 5 (a lot), relationship satisfaction ranged from 1 (not at all) to 5 (very much), allostatic load scored from 0 (very low) to 4 (very high), gender was coded 0 = men, 1 = women*.

a *language of study participation was coded 1 = English and 0 = Mandarin*.

All continuous variables were grand mean-centered. Due to parsimony, education was excluded as a control variable because it was not significantly related to any of the outcome variables

b Based on external rating

* *p < 0.05*.

** *p < 0.01*,

*** *p < 0.001*.

### Allostatic Load (Hp2)

Hp2 was examined using a similar multilevel model (Table 3). There were positive main effects for self-rated health (*b* = −0.13, *SE* = 0.06, *p* = 0.040) and English as language of study participation (*b* = 0.27, *SE* = 0.13, *p* = 0.040). No main effects for joint goals and over-reporting were found. However, the hypothesized interaction between the number of joint goals and over-reporting was significant (*b* = 0.18, *SE* = 0.09, *p* = 0.033; Figure 1). Simple slope analysis indicated that a high number of joint goals in combination with low over-reporting was associated with lower allostatic load; the simple slope for low over-reporting (1 *SD* below the mean) was −0.20 (0.07), *t* = −3.05, *p* = 0.003; the simple slope for average over-reporting (at mean value) was −0.02 (0.07), *t* = −0.35, *p* = 0.723 and the simple slope for high over-reporting (1 *SD* above the mean) was 0.15 (0.12), *t* = 1.28, *p* = 0.201. The model explained 15.89% of the variance.

### Relationship Satisfaction (Hp3)

Better self-rated health (*b* = 0.17, *SE* = 0.04, *p* < 0.001), being a man (*b* = −0.18, *SE* = 0.07, *p* = 0.007), and English as language of study participation (*b* = 0.37, *SE* = 0.96, *p* < 0.001) were associated with higher relationship satisfaction. There was a significant main effect for the number of externally-rated joint goals (*b* = 0.16, *SE* = 0.09, *p* = 0.016), and for over-reporting (*b* = 0.13, *SE* = 0.05, *p* = 0.009), indicating that participants were more satisfied with their relationship if they over-reported and had more externally judged joint goals^3^. The overall model explained 51.91% of the variance.

## Discussion

This brief report investigated how joint goals are related to goal progress, allostatic load, and relationship satisfaction. Findings indicate that a high proportion of participants' three most salient goals were joint (external rating 30.9%, self-report 60.1%). Comparing participants' self-reports with external ratings of joint goals points to systematic and potentially meaningful differences. About two-thirds of participants over-reported at least one joint goal (thinking their goal was joint without the partner mentioning it among their three most salient goals). Results show that the number of externally-rated joint goals in combination with little over-reporting was associated with more goal progress and lower allostatic load. Having many joint goals and over-reporting were each related to higher relationship satisfaction.

### Joint Goals and Goal Progress (Hp1)

In line with Hp1, we showed that a high number of joint goals was related to more goal progress 1 week later, but only for participants with low over-reporting. By comparing both partners' goals, we were able to disentangle the effects of joint goal perceptions and over-reporting and uncovered unique associations with goal progress. This finding is consistent with the idea that it is important to know a partner's goals to engage in goal coordination. According to the Transactive-Goal-Dynamics Theory (Fitzsimons and Finkel, 2018), goal coordination is a key factor for translating joint goals into goal progress. Inaccurate perceptions of joint goals could waste energy when trying to get a partner involved in progress on a goal they do not care about. Ultimately, this could lead to frustration and undermine goal-relevant efforts. Possible mechanisms behind the observed moderation of joint goals and over-reporting might be more effective collaborative problem-solving (Hoppmann and Gerstorf, 2013) or higher frequency and enjoyment of collaboration (Schindler et al., 2010).

### Allostatic Load (Hp2)

In line with Hp2, joint goals were related to better individual health if perceived accurately. We also found that the highest proportion of joint goals appeared in the health and physical activity domains. This is consistent with propositions that health behaviors may be important variables linking psychosocial resources with allostatic load (Wiley et al., 2017). Goal coordination (Fitzsimons and Finkel, 2018), joint implementation of goal-directed activity (Berli et al., 2018), and dyadic planning (Keller et al., 2017) all require knowledge of a partner's goals and have been linked to health behavior engagement. Interpreting the moderation effect, we assume that an accurate perception of the partners' salient goals (little over-reporting) facilitates jointly coordinating goal-directed activity. Importantly, no causal conclusions can be drawn from our findings. For example, Wiley et al. (2017) argue that the reverse is possible: high allostatic load could undermine psychosocial resources, for example, by acting as a stressor.

### Relationship Satisfaction (Hp3)

In line with Hp3, a larger number of joint goals was associated with higher relationship satisfaction. One possible linking variable could be trait similarity: joint goals are more common in couples with similar traits (Gray and Coons, 2017), and trait similarity among partners, in turn, has been linked with high relationship satisfaction (e.g., Malouff et al., 2010). Notably, the reverse (relationship satisfaction predicting joint goals) could be true as well and needs to be tested using more mechanism-oriented longitudinal study designs.

An unexpected result relates to the fact that more over-reporting was associated with higher relationship satisfaction. There has been a debate about whether it is necessary to view the world accurately or if it might sometimes be adaptive to have positively biased views. In this sense, over-reporting could be interpreted as an indicator of positive illusions. Seminal work by Taylor and Brown (1994) argues that positive illusions have positive effects on well-being. Our results linking over-reporting with relationship satisfaction are consistent with this idea. Importantly, the divergent findings regarding goal progress and allostatic load (in interaction with the number of joint goals) are in line with Taylor's and Brown's (1994) proposition that positive illusions do not have to be unanimously positive and that, in fact, they can backfire. It might be that joint goals and positively biased perceptions capture processes that are essential for satisfying relationships; however, not having an accurate reading of a partner's goals may undermine collaborative efforts to work toward goals and play a role in allostatic load, e.g., through poorer health behaviors (Kaul and Lakey, 2003).

### Implications of Findings

So what shall we tell older adult couples? It depends on what is most important for a given couple in a specific situation: that they make progress on their goals, that they optimize their health, or that they happily live together. This is in line with previous research showing that consensus between couples differs in its effect on distress depending on the area of consensus (Reyes et al., 2020). If being satisfied with their relationship is the priority, biased perceptions might not be so bad (Story et al., 2007).

There is initial evidence that addressing health behavior change in both partners of a couple has favorable outcomes (Jackson et al., 2015; Richards et al., 2018). Our results further underpin the notion that health interventions should capitalize on significant others.

If the importance of joint goals is corroborated in future studies, it will be essential to inform older adults about the meaning of joint goals. It would be interesting for future studies to develop interventions showing partners how they can turn “me goals” into “we goals” and to facilitate translating them into action, for example, through dyadic behavior change techniques (Knoll et al., 2017).

To give a broader outlook, integrating goal setting and progress discussions into patient care plans is an increasing need in medicine (Schulman-Green et al., 2006). The findings of this study suggest that including the patient's partner in the goal-setting process poses a crucial step to be considered in all efforts of improving patient-centered care.

### Limitations

Our goal assessment has some degree of ambiguity. We focused on three spontaneously generated particularly salient goals. It is possible that when one partner reported their goal as joint, but the other partner did not list it, that it could have shown up further down the list. Also, a focus on only three goals restricts variability. However, as goals represent a very complex system and people often pursue multiple personal goals across various life domains which might compete with each other (Kruglanski et al., 2002; Riediger and Freund, 2004; Presseau et al., 2013), a “complete list of their individual and joint goals” can probably never be reached. Nevertheless, focusing on particularly salient goals should be seen as a starting point that warrants further extension. In addition, we do not distinguish between partners sharing a goal and wanting to achieve it together. However, with this qualitative assessment of goals, we were able to assess and rate individually generated personal goals without imposing restrictions on their content.

Of note, the effects of joint goals and over-reporting could be different in younger adults, recently married older adults and adults living with health problems. For example, as goal pursuit might be particularly difficult for adults living with significant health problems, this population might especially benefit from joint goals and collaborative problem solving (Schindler et al., 2010).

Furthermore, data analytic choices were made conceptually and had to consider power limitations related to the sample size (118 couples). We hope that future work with larger samples builds on our findings and extends them by estimating actor and partner effects using SEM approaches.

Lastly, we cannot draw conclusions about the underlying mechanisms. Recent research on dyadic self-regulatory processes such as dyadic planning (Knoll et al., 2017), couple self-efficacy, and communal coping (Lewis et al., 2006) might be starting points. Finally, no causal conclusions can be drawn based on our correlational findings. To address these limitations, a follow-up study assessing goals more comprehensively and experimentally manipulating potential underlying factors by teaching different dyadic goal setting and behavior change strategies would be valuable.

In conclusion, when studying older adults' goals, it is essential to include the partner because our results show that older adults want to achieve a high proportion of their goals together as a team. A high number of joint goals appears to have positive ramifications for diverse outcomes such as goal progress, allostatic load, and relationship satisfaction.

## Data Availability Statement

The raw data supporting the conclusions of this article will be made available by the authors, without undue reservation.

## Ethics Statement

The studies involving human participants were reviewed and approved by Clinical Research Ethics Board of the University of British Columbia. The patients/participants provided their written informed consent to participate in this study.

## Author Contributions

CH, MA, DG, and KM designed and directed the project and supervised the work. NU performed the data analysis and the goal rating and wrote the manuscript. VM managed the project, recruited participants, performed the study, and did the data management. SB performed the goal rating and assisted with drafting the manuscript. TP contributed to the data analysis and discussing the results. All authors commented on the manuscript and contributed to the final version of the manuscript.

## Conflict of Interest

The authors declare that the research was conducted in the absence of any commercial or financial relationships that could be construed as a potential conflict of interest.

## Footnotes

^1^“No over-reporting” mostly means there was consensus between self-report and external rating. Only in a minority of goals (5.9–11.1% for each of the three goals), the external rating but not the self-rating was joint.

^2^Thirty couples were not asked to provide saliva samples because of thyroid dysfunction.

^3^Calculating all main-analyses without the control variables yielded the same result patterns, see Supplementary Material 4.
