# Supplementary material for: Joint Goals in Older Couples: Associations With Goal Progress, Allostatic Load, and Relationship Satisfaction
Source: Front Psychol. 2021 Apr 20;12:623037. doi: 10.3389/fpsyg.2021.623037 (PMC8093431; doi:10.3389/fpsyg.2021.623037)
Supplement: Supplementary file 1 [file Data_Sheet_1.pdf]

Supplementary Material – S1

1 Procedure

The study was part of the larger project on spousal health dynamics (also described in Pauly et al., 2019). This project examines relationship-, individual-, and situation-specific factors for shaping psychological well-being, health behaviors as well as longer-term health outcomes.

Couples aged 60 years and above were recruited in the greater Vancouver area using various strategies (e.g., print media, online forums, community organizations). Given the local demographics this study was conducted in English and Cantonese/Mandarin depending on the participants' primary language. Informed consent was obtained from all research participants (University of British Columbia ethics board). Each partner received $100 compensation. The study was powered and financed to recruit 120 couples with complete datasets. This manuscript answers a secondary research question, so it was not included in the power analysis. From the original 258 participants who entered the study, nine couples dropped out after the baseline session. Two further couples had to be excluded due to missing values in the main outcome variables. Participants included in the analyses were 118 community-dwelling couples (N = 236 individuals).

Participants took part in a three-hour, in-person baseline session. Couples completed various individual difference measures (including assessments of goals and demographics) independently of each other. Additionally, researchers assessed different health indices (blood pressure, weight, height, waist-to-hip ratio). Afterward, a 1-week time-sampling phase took place. During this time-sampling phase, participants completed brief electronic surveys and provided saliva samples five times per day using Salivettes (Sarstedt, Germany) for the allostatic load measure. Finally, couples were invited to a 2-hour in-person exit session where they completed several questionnaires and cognitive tasks (including goal progress and relationship satisfaction ratings). Participants also provided a blood sample at a community lab near their residence (for the measure of allostatic load). There was also a later long-term longitudinal component to this study.

2 Statistical analyses

For the main analyses, hierarchical linear models were conducted using the R package lme4 (Bates et al., 2014). A 2-level random intercept model accounted for the nested data structure in the sample: individuals (level 1) nested within couples (level 2). Outcome variables of the three models were (1) goal progress, (2) relationship satisfaction, and (3) allostatic load. Predictor variables were (1) the number of joint goals (externally-rated) at the couple-level, (2) the number of over-reporting at the individual-level, and (3) the interaction between the two. The interaction was decomposed by calculating simple slopes (Preacher et al., 2006). Education (some versus no university education), gender, age, language of study participation (English vs. Mandarin), and self-rated health ("poor=1" to "excellent=5") were considered as control variables. For reasons of parsimony, education was excluded as a control variable because it was not significantly related to any of the outcome variables. Random slopes were left out of the model due to parsimony (Campbell and Kashy, 2002). All continuous

variables were grand-mean centered. Full maximum likelihood estimation was used. Explained variance R^2 is reported using the R package MuMIN (Barton, 2009).

Bates, D., Mächler, M., Bolker, B., and Walker, S. (2014). Fitting linear mixed-effects models using lme4. *Journal of Statistical Software* 67(1). doi: 10.18637/jss.v067.i01.

Campbell, L., and Kashy, D.A. (2002). Estimating actor, partner, and interaction effects for dyadic data using PROC MIXED and HLM: A user–friendly guide. *Personal Relationships* 9(3)**,** 327-342. doi: 10.1111/1475-6811.00023.

Pauly, T., Michalowski, V.I., Nater, U.M., Gerstorf, D., Ashe, M.C., Madden, K.M., et al. (2019). Everyday associations between older adults' physical activity, negative affect, and cortisol. *Health Psychology* 38(6)**,** 494-501. doi: 10.1037/hea0000743.

Preacher, K.J., Curran, P.J., and Bauer, D.J. (2006). Computational tools for probing interactions in multiple linear regression, multilevel modeling, and latent curve analysis. *Journal of Educational and Behavioral Statistics* 31(4)**,** 437-448. doi: 10.3102/10769986031004437.
