# Supplementary material for: Joint Goals in Older Couples: Associations With Goal Progress, Allostatic Load, and Relationship Satisfaction
Source: Front Psychol. 2021 Apr 20;12:623037. doi: 10.3389/fpsyg.2021.623037 (PMC8093431; doi:10.3389/fpsyg.2021.623037)
Supplement: Supplementary file 2 [file Data_Sheet_2.pdf]

frontiers

Supplementary Material – S2

1 Personal Goals Questionnaire

We are interested in the kinds of activities and concerns that people have at different stages of their lives. We call these **personal projects**. All of us have a number of personal projects at any given time that we **think about**, **plan for**, **carry out**, and sometimes (though not always) **complete.**

Personal projects can be related to many different life domains such as partnership, family, friends, health, memory, leisure, finances, work etc.

We are particularly interested in those projects…

…that you are planning to actively pursue within the **upcoming weeks**

…whose realization is highly important for you **right now**

…that influence your **daily** life and the **activities** in which you engage

Please take a moment to think about your personal projects and write them down below.

These are the projects I plan to pursue within the upcoming weeks:

GOAL A: ____

____

____

____

____

GOAL B: ____

____

____

____

____

GOAL C: ____

____

____

____

____

Please take a moment to write down your personal projects onto the attached **Goal Card** for use during the rest of the project.

Next, we are interested in finding out a little bit more about the roles these goals play for you at this moment. We will ask several questions **<u>with respect to each of these goals separately</u>**.

Please place your **Goal Card** next to you on the table in order to be able to refer back to your goals.

A. With respect to Goal A:

1. Which life domain does this goal belong to? (select <u>ALL</u> that apply)

______ partnership

______ family

______ friends

______ physical activity

______ volunteering

______ finances

______ cognition or memory

______ health

______ work/productive activities

______ home management

______ leisure

______ other

…

8. Is this a goal that you and your partner have in common and want to achieve together?

1. Yes
2. No

B. With respect to Goal B:

1. Which life domain does this goal belong to? (select ALL that apply)

______ partnership	______ cognition or memory
______ family	______ health
______ friends	______ work/productive activities
______ physical activity	______ home management
______ volunteering	______ leisure
______ finances	______ other

…

8. Is this a goal that you and your partner have in common and want to achieve together?

1. Yes
2. No

- Not at All
- A Little
- Moderately
- Quite a Bit
- Very Much

C. With respect to Goal C:

1. Which life domain does this goal belong to? (select ALL that apply)

______ partnership

______ family

______ friends

______ physical activity

______ volunteering

______ finances

______ cognition or memory

______ health

______ work/productive activities

______ home management

______ leisure

______ other

…

8. Is this a goal that you and your partner have in common and want to achieve together?

1. Yes
2. No
