# Supplementary material for: Joint Goals in Older Couples: Associations With Goal Progress, Allostatic Load, and Relationship Satisfaction
Source: Front Psychol. 2021 Apr 20;12:623037. doi: 10.3389/fpsyg.2021.623037 (PMC8093431; doi:10.3389/fpsyg.2021.623037)
Supplement: Supplementary file 3 [file Data_Sheet_3.pdf]

Supplementary Material – S3

1 Coding scheme: joint versus individual goals

Do both partners mention the same goal?

1 = yes, both partners mention this goal

0 = no, this goal was mentioned by one partner only

General rules:

1. It is not relevant if the goal is mentioned as goal A, B or C
   - (e.g. partner 1 mentions "more walking" as project A, partner 2 mentions "more walking" as project C → still rate it as 1)
2. The wording of the goal does not matter, only the content
   - (e.g. partner 1 says "I want to eat a healthy breakfast", partner 2 says „eating something healthy in the morning" → still rate it as 1)
3. Check, if the two goals were classified by the two partners into the same category (variables [ga1a_bs] :[ga1l_bs] such as "health", "family", "work", etc.). Fill out the variable "category_right" (Do the goals belong to (at least one) same category reported by the participants? → 1=yes; 0=no).
4. If you are not sure about your rating, the participant's rating can be helpful for your decision (e.g. rate a weight loss-goals and an exercise goal as joint, if the weight loss-goal is rated as "physical activity" by the participant)
5. If the goal is connected to more than one goal of the partner just report the goal with the strongest connection.

Rating details:

a) If both partners mention the same third person in their goal (e.g. grandson, daughter, the same name, etc.), rate it as 1 (=same goal). This also applies, when the activity they want to do with this person, is not exactly the same and the time-frame or place differs. This also applies if one partner mentions more than one other person.
   - (e.g. all of the following goals should be rated das 1 (=same)
   - "spend more time with my grandson"
   - "showing my grandson how much I love him"
   - "read more books with my grandson at home"
   - "play with my grandson at the playground every week"
   - "helping my daughter, my son and my grandson"
   - HOWEVER: this does not apply, if the goals describe distinct activities that are definitely not the same (see e).
   - e.g. all of the following goals should be rated das 0 (= not the same)
   - "teach my grandson how to play the piano at least twice a week"

- “play with my grandson at the playground every week“
- “go to the gym with my grandson “
-

b) If both partners mention the same activity (e.g. holiday, concert) rate it as 1 (=same goal). This also applies, if there is different reasoning for it, different circumstances, different time frames, etc.
    - e.g. all the following goals should be rated as 1 (=same)
    - “I want to postpone my surgery to go on holiday“
    - “planning trips for my holiday“
    - “staying healthy so I can go on holiday“
    - OR
    - “tidying up the room“
    - “tidying up the room every evening“
    - “having always a tidy room“

    HOWEVER: this does not apply, if the goals describe distinct activities that are definitely not the same (see e).

c) If one activity describes a sub-activity of the other, still rate it as 1 (= same goal)
    - (e.g. the following 2 goals should be rated das 1 (=same)
    - “doing more exercise“
    - “going for a run more regularly“
    - OR
    - “doing housework“
    - “doing the laundry“
    - OR
    - “spending more time at the computer“
    - “learning how to use the internet“

d) If one category describes the same content, but goes more into details, still rate it as „1“ (= same goal)
    - e.g. all these goals should be rated das 1 (=same)
    - “being thankful“
    - “thinking every evening of the three most pleasant events/contacts and appreciating them“

e) If the goals described are distinct activities that are definitely not the same, rate them as 0 (= not the same goal). This also applies if they fall under the same category.
    - e.g. all of the following goals should be rated das 0 (=not the same)
    - “go for a run more often“
    - “play table tennis more often“
    - “swimming regularly“

f) It does not matter if both partner can do the goal together (e.g. both say playing tennis), or not (first example : both say “being more engaged in my job“;
    - Second example : partner 1 says “supporting partner 2 to eat healthier“ and partner 2 says “I want to eat healthier”). Both can be joint goals.
