# Supplementary material for: Joint Goals in Older Couples: Associations With Goal Progress, Allostatic Load, and Relationship Satisfaction
Source: Front Psychol. 2021 Apr 20;12:623037. doi: 10.3389/fpsyg.2021.623037 (PMC8093431; doi:10.3389/fpsyg.2021.623037)
Supplement: Supplementary file 4 [file Data_Sheet_4.pdf]

frontiers

Supplementary Material – S4

Table S4

Multilevel analysis without control variables for goal progress, relationship-satisfaction, and allostatic load

	Goal Progress				Allostatic Load				Relationship Satisfaction			
	Coefficient (*b*)		*SE*	*p*-value	Coefficient (*b*)		*SE*	*p*-value	Coefficient (*b*)		*SE*	*p*-value
**Intercept**	3.032	***	0.069	<.001	1.141	***	0.065	<.001	4.137	***	0.059	<.001
**Level 1 (person)**												
Over-reporting	-0.012		0.080	.882	-0.044		0.085	.552	0.148	**	0.052	.005
**Level 2 (couple)**												
Number of joint goals[a]	-0.052		0.093	.572	0.009		0.087	.922	0.109		0.075	.148
**Interaction**												
Over-reporting x number of joint goals	-0.214	*	0.093	.022	0.194	*	0.087	.026	-0.099		0.066	.137

Note. N =118 couples; *SE* = standard error, goal progress ranged from 1 (none) to 5 (a lot), relationship satisfaction ranged from 1(not at all) to 5 (very much), allostatic load scored from 0 (very low) to 4 (very high). All continuous variables were grand mean-centered. [a]Based on external rating

* $p < .05$. ** $p < .01$, *** $p < .001$
